# A Novel Phylogenetic Negative Binomial Regression Model for Count-Dependent Variables

**DOI:** 10.3390/biology12081148

**Published:** 2023-08-19

**Authors:** Dwueng-Chwuan Jhwueng, Chi-Yu Wu

**Affiliations:** Department of Statistics, Feng-Chia University, Taichung 407, Taiwan

**Keywords:** phylogenetic comparative analysis, trait evolution, Poisson regression, negative binomial regression, generalized estimating equation

## Abstract

**Simple Summary:**

Researchers identified a challenge in analyzing count-dependent variables in species related through a shared ancestry using traditional regression models, as these models often overlook the inherent interdependence from common lineage. To address this, a new phylogenetic negative binomial regression model was developed that recognizes this lineage dependence and allows for overdispersion, surpassing the limitations of the conventional generalized linear models (GLMs). Using the generalized estimating equation (GEE) framework, this model offers precise parameter estimation. This innovation offers a more accurate analysis tool for understanding species data, emphasizing the influence of shared ancestry and promises enhanced research methodologies, bringing valuable perspectives to the fields of evolutionary biology and ecology.

**Abstract:**

Regression models are extensively used to explore the relationship between a dependent variable and its covariates. These models work well when the dependent variable is categorical and the data are supposedly independent, as is the case with generalized linear models (GLMs). However, trait data from related species do not operate under these conditions due to their shared common ancestry, leading to dependence that can be illustrated through a phylogenetic tree. In response to the analytical challenges of count-dependent variables in phylogenetically related species, we have developed a novel phylogenetic negative binomial regression model that allows for overdispersion, a limitation present in the phylogenetic Poisson regression model in the literature. This model overcomes limitations of conventional GLMs, which overlook the inherent dependence arising from shared lineage. Instead, our proposed model acknowledges this factor and uses the generalized estimating equation (GEE) framework for precise parameter estimation. The effectiveness of the proposed model was corroborated by a rigorous simulation study, which, despite the need for careful convergence monitoring, demonstrated its reasonable efficacy. The empirical application of the model to lizard egg-laying count and mammalian litter size data further highlighted its practical relevance. In particular, our results identified negative correlations between increases in egg mass, litter size, ovulation rate, and gestation length with respective yearly counts, while a positive correlation was observed with species lifespan. This study underscores the importance of our proposed model in providing nuanced and accurate analyses of count-dependent variables in related species, highlighting the often overlooked impact of shared ancestry. The model represents a critical advance in research methodologies, opening new avenues for interpretation of related species data in the field.

## 1. Introduction

Phylogenetic comparative methods (PCMs) have a well-established history of illuminating the underpinnings of trait evolution, leveraging the rich insights present within phylogenetic trees [1]. They have traditionally been employed in the analysis of quantitative trait evolution, a practice deeply ingrained in the academic literature [2,3,4,5,6]. Despite this, an emerging and consistent observation within this area of study is the evolution of categorical traits, which are often represented in categorical or count forms and extend across diverse species. The vast applicability of these methods in a variety of biological scenarios underscores their importance, not only for specialists but also for a broader community of researchers.

Count data has been successfully used to elucidate a range of biological phenomena, for example, the number of toxicological activities in snake venom, or functional activities [7]. In this study, Poisson regression models identified the diversity of diet as a significant predictor of venom’s functional activity, demonstrating the value of such models in making ecological predictions. Similarly, in a study on Amazonian forest birds, count data played a crucial role in unraveling the relationship between body mass, flight efficiency, diet, and road-crossing frequency [8]. Here, binomial regression models provided valuable insights into the predictors of road-crossing, which serves as a proxy for the bird’s ability to cross habitat gaps—an essential survival skill in the rapidly changing Amazonian landscape. Furthermore, count data in the form of gene copy numbers in yeast species has been utilized to investigate the relationship between metabolic gene copy number and growth rate. A comparative analysis using GEE [9] revealed a clear correlation, providing significant insights into yeast ecology. However, the challenge in such studies often lies in the appropriate analysis of count data. Traditional linear regression forms are ill-suited for such data, since the assumption of normality in the residuals can lead to misleading results when applied to count values.

Hence, there arises the need for alternative models that can adequately account for the specific nature of count data. Enter the realm of GLMs, which includes the phylogenetic Poisson regression [9] and the phylogenetic negative binomial regression which will be developed in this study, that serve as robust tools for such data. Both these models consider the count nature of the data but differ in their assumptions. While the Poisson regression model assumes equal mean and variance, the negative binomial regression model is equipped to handle overdispersion, where the variance exceeds the mean. Although both models find use in different scenarios, it is crucial for practitioners to be aware of potential inaccuracies resulting from the Poisson regression model if the assumption of equal mean and variance is violated [10]. In such scenarios, the phylogenetic negative binomial regression model presents itself as a superior alternative, offering an extra parameter to independently adjust the variance from the mean. This independence can improve model fit and provide more accurate results, highlighting the model’s significance.

Furthermore, while the application of the Poisson regression framework is well detailed in previous studies [9], our work focuses on the novel application of the negative binomial regression model in the context of phylogenetic regressions. The remainder of this study, therefore, seeks to introduce this novel phylogenetic negative binomial regression model, test it rigorously, and demonstrate its utility in analyzing count-dependent variables. We believe that the insights gained from this endeavor will provide a fresh perspective to researchers in trait evolution and related fields, enabling a more comprehensive and nuanced understanding of evolutionary dynamics. We will demonstrate the model’s efficacy through two distinct empirical assessments: an analysis of lizard egg count as it relates to body mass, and an exploration of mammalian litter size influenced by factors such as the number of teats, longevity, body mass, etc. Through these applications, we hope to underscore the model’s utility and contribute to improved methodologies in the study of related species.

The paper is structured as follows: Section 2 outlines our methodology. Section 2.1 discusses regression under a GLM framework, specifically delving into independent Poisson and negative binomial regression. In Section 2.4, we elaborate on the regression under a GEE for phylogenetically dependent data, emphasizing GEE for phylogenetic Poisson and negative binomial regression. Section 3.2 documents our empirical studies on lizard egg-laying and mammalian litter sizes. We present the results of our work, including simulation and empirical analysis outcomes, in Section 3. This is followed by the discussion in Section 4, and the conclusion in Section 5. Scripts and relevant files developed for this project can be accessed in https://www.tonyjhwueng.info/phypoinb2reg accessed on 27 July 2023.

## 2. Materials and Methods

We present the regression models utilized for analyzing count variables. Traditional linear regression methods are often inadequate for handling count data analysis, primarily due to their assumption of normally distributed residuals, which is unsuitable for count data. In a setting independent of evolution for a group of species, the regression analysis using count data as the response variable and other covariates is analyzed using the GLM described in Section 2.1 where the Poisson regression is described in Section 2.1.1 and the negative binomial regression is described in Section 2.1.2. Note that [11] considered using a single predictor for modeling the count variable under a negative binomial regression model for a couple of empirical data analyzes, our study proposes a general framework concerning multiple covariates and provides a detailed inference. When considering evolution as a dependent process described by a phylogenetic tree relation among species, the regression analysis using count data as response variables and other covariates is carried out by the generalized estimation equation (GEE) in Section 2.4 where the phylogenetic Poisson regression is described in Section 2.4.1 and the phylogenetic negative binomial regression is described in Section 2.4.2.

### 2.1. Applying GLM in Regression Analysis

GLMs are fundamental tools for regression analysis across various scientific fields, including biology. They offer a flexible statistical framework to analyze different types of response variables, making them an invaluable tool in the biological researcher’s toolkit. In the following subsections, we delve into two specific applications of GLMs in the context of biological research: independent Poisson regression and independent negative binomial regression.

The first Section 2.1.1 elaborates on the use of independent Poisson regression, a powerful method particularly suited for the analysis of count data, which is frequently encountered in biological studies. Subsequently, in Section 2.1.2, we turn our attention to negative binomial regression, a model instrumental in handling count data exhibiting overdispersion—a common phenomenon in biological data. Details of the models’ mathematical formulation can be found in Appendix A.3, specifically in Appendix A.3.1 and Appendix A.3.2.

#### 2.1.1. Independent Poisson Regression in Biology

Biological research often calls for the analysis of count data—be it bacterial colonies in a dish [12], the number of times a gene gets expressed [13], or species enumerated in an ecological survey [14]. A method conducive to such an analysis is Poisson regression, an efficient instrument to evaluate count data [15]. This technique assumes that the response variable, adheres to a Poisson distribution suitable for count variables, with a mean occurrence rate λ. The probability mass function of the Poisson random variable *y* is

(1) $$f\left(y|\right)=\frac{\exp\left(-\lambda \right)\lambda^{y}}{y!},y=0,1,2,\cdots \cdots .$$

Poisson regression applies a log link function, making it suitable for count data analysis and potentially providing more reliable statistical outcomes [16]. To determine parameters in a Poisson regression model, one can utilize the maximum likelihood estimation (MLE) method, employing numerical strategies such as Newton’s method for deriving the MLE [17] (see Appendix A.3.1).

#### 2.1.2. Exploiting Negative Binomial Regression for Overdispersion

In biological studies, researchers frequently confront situations where the response variable is count-based and variable in a way that it surpasses the mean. This phenomenon, called overdispersion, suggests an inherent data structure that requires careful modeling. In these instances, the negative binomial regression model becomes an instrumental tool for analysis in various biolofical fields such as the molecular count data from scRNA-seq experiments [18], the weekly dengue haemorrhagic fever cases [19], or  the number of fledglings from a nest or inflorescences on a plant [20].

The negative binomial distribution adds an additional parameter (often denoted as *r*) which models the over-dispersion relative to the Poisson distribution (where the mean equals the variance). This is particularly useful for count data, where often the variance is greater than the mean. The negative binomial model operates under the assumption that the response variable follows a negative binomial distribution. The probability mass function of the negative binomial random variable *y* is

(2) f(y|p,r)=y+r−1ypr(1−p)y,y=r,r+1,r+2,⋯

where 0≤p≤1 is the probability of success. The model establishes a relationship between the mean response and its predictors through a logarithmic link function, creating a linear relationship with the parameters [17]. This mathematical framework suggests that a systematic alteration in a predictor variable leads to a proportional change in the response. Further details on this can be found in Appendix A.3.2.

In Section 2.2 we provide a preliminary analysis for two empirical datasets using the two count regression models of independent types.

### 2.2. A Preliminary Analysis

A quick analysis of two empirical datasets using the two GLMs is reported in Table 1 where two fitted regression models (GLM: Poisson regression model vs. the negative binomial model) for the lizard dataset and the mammal dataset are presented. The response variable for the lizard dataset [21] is the egg number per year (EPY) with the covariates egg mass (EM) in gram. The response variable for the mammal dataset [22] is the litter number per year (LY) with another 4 covariates: litter body mass (LS), offspring value as per equation (OV), longevity in years (LG), and whether at least 1 established alien population has successfully spread or not (Spread).

For the mammalian dataset, the variance (2.37) slightly surpasses the mean (1.99), favoring the Poisson regression model, as evidenced by a lower AICc value and a higher weight compared to the negative binomial regression model. In contrast, for the lizard dataset, the variance (55.03) significantly exceeds the mean (20.82) in egg count per year. This discrepancy favors the negative binomial regression model, which has a lower AICc value and a higher weight compared to the Poisson regression model. This preference for the negative binomial model may be attributed to its unique ability to handle overdispersion, a feature where the phylogenetic negative binomial model particularly excels.

The AICc [23], defined in Equation (3), provides a measure for comparing the quality of different statistical models for a dataset.

(3) $$\mathrm{AICc}=\mathrm{AIC}+\frac{2k(k+1)}{n-k-1}.$$

Here, AIC is the Akaike Information Criterion ($2k-2\ln(\hat{L})$), *k* is the number of parameters, $\ln(\hat{L})$ is the likelihood value computed from using parameter estimates, and *n* is the taxa size. The Akaike weights $w_{i}$ for the *i*th model measured the importance of the models in the set of candidate models are calculated using Equation (4):

(4) $$w_{i}=\frac{\exp\left(-\frac{1}{2}\Delta_{\mathrm{AICc},i}\right)}{\sum_{j=1}^{m}\exp\left(-\frac{1}{2}\Delta_{\mathrm{AICc},j}\right)},$$

where $\Delta_{\mathrm{AICc},j}={\mathrm{AICc}}_{j}-\min_{1\leq j\leq m}\left\{{\mathrm{AICc}}_{j}\right\}$ [24] represents the difference in AICc values between model *j* and the model with the smallest AICc value (the best model among *m* models) and provides a measure of how much worse model *j* is compared to the best model. Here, i=1,2 where i=1 for Poisson regression and i=2 for negative binomial regression.

In this equation, $\Delta {\mathrm{AICc}}_{i}$ is the difference in AICc values between the *i*th model and the minimal AICc model. The comparison of the fit using the modified Akaike Information criteria (AIC) [25] is shown in Table 1 where the two empirical datasets show a slight preference for either model. For the mammal dataset, the response trait (litter number) has a mean 1.986 and a variance of 2.370. The Poisson regression model provides a slightly better fit to this dataset. For the lizard dataset, the response trait (egg count per year) has a variance of 55.029 and a mean of 20.824. In addition, the regression analysis using covariates: size at maturity, average size, age at maturity, egg mass, clutch size, and clutch mass favors the negative binomial regression model over the Poisson regression model.

In Section 2.3, we introduce the phylogenetic trait evolution of both continuous types as well as the discrete types associated with their count regression model.

### 2.3. Phylogenetic Trait Evolution

**Figure 1:**
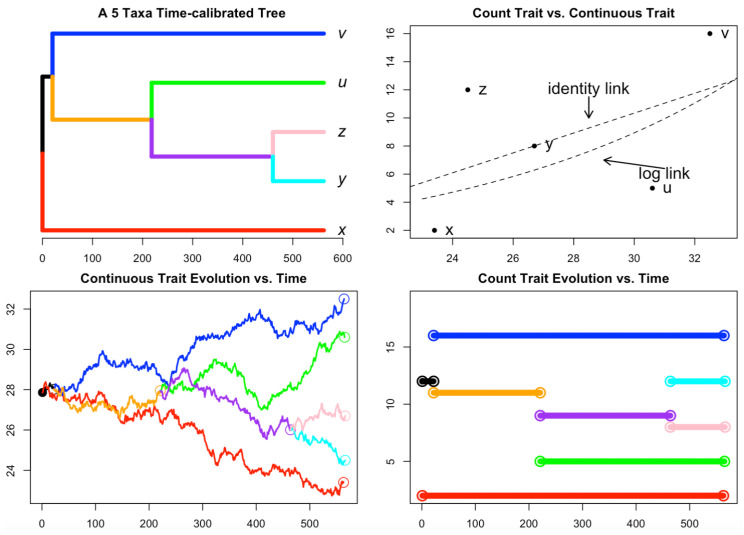
Tree, count trait process, quantitative process, and the bivariate scatter plot. Upper-left panel: a phylogenetic tree of 5 taxa v,u,z,y,x. Upper-right panel: a scatter plot for the count trait data vs. continuous trait data for the 5 taxa. Lower-right panel: a count trait evolution along the phylogenetic tree dependence following a randomized counting process (not necessarily non-decreasing). Lower-left panel: a quantitative trait evolution along the phylogenetic tree with trait values. Each color in the image corresponds to a different branch. The tip values for both the count trait and the continuous are reported in the scatter plot in the upper-right panel. The C matrix corresponding to the tree is shown in Equation (5).

It has been widely accepted that due to speciation and other evolutionary phenomena, species evolved in a dependent manner along a phylogenetic tree. The regression analysis may be more robust when incorporating trees into the analysis. For instance, a five-species phylogenetic tree containing 5 taxa v,u,z,y, and *x* is presented in Figure 1.

For the continuous trait evolution shown in the lower right panel of Figure 1, trajectories are simulated using the tree traversal algorithm under a continuous random process [26] where five speciation events have occurred in subsequent order, starting at the root (t=0) and continuing immediately afterward. The observed trait values (comparative data) for these five species, represented by $v_{t},u_{t},z_{t},y_{t}$, and $x_{t}$, are captured at t=570.

The evolution of these traits can be described using the Brownian motion model (BM) [27]. As an example, the trait variable for species *v*, for example, observed at time *t*, is expressed as $v_{t}=\rho +\sigma_{v}W_{t}^{v}$. Here, $\rho =v_{0}$ denotes the ancestral state of species *v*, $\sigma_{v}$ represents a positive constant parameter, which is the rate of evolution, and $W_{t}^{v}$ is a Wiener process, a mathematical construct used in the modeling of stochastic processes. Each species is assumed to have the same rate $\sigma_{i}=\sigma$, for i=v,u,z,y,x and possess independent identical Wiener processes $W_{t}^{i}=W_{t}$, for i=v,u,z,y,x.

For the count trait evolution shown in the lower-left panel of Figure 1, The tips values at t=570 denoted as (x,y,z,u,v) are assumed to have values Y=(2,8,12,5,16). Note that one can also consider generating the sample through a tree traversal [28] where starting with the root node with a given value then each successive internal node (the circled points in the figure) is simulated using the status of the starting node plus or minus a Poisson random variable with the rate equal to the branch length multiplied by the status of the nodes where the plus or minus is determined by a Bernoulli trial with value 1 or −1 with probability drawn from a uniform distribution.

It has been known that the tree is incorporated into the analysis for quantitative regression analysis and many packages have been developed to contribute to the community [29,30,31,32]. However, conceiving that the negative binomial regression may be potentially useful to analyze count data in phylogenetic regression analysis as the Poisson regression, this work delineates the two phylogenetic regression models for counting dependent variables in a more comprehensive manner using simulation and empirical analysis. In particular, the C matrix will be used for modeling the dependent relationship for the phylogenetic regression using the count response variable. Since the tree can be equivalently transformed into a square matrix C where each element of $c_{ij}\in C$ measures the shared branch length between the two tips [33,34]. For example, the C for the tree in in the upper left panel of Figure 1 can be represented as in Equation (5).

(5) $$\begin{array}{ccc} & & \begin{array}{cccccc} \;x\; & y\; & z\; & u\; & v & \end{array}\\ \begin{array}{c} C= \end{array} & \begin{array}{c} x\\ y\\ z\\ u\\ v \end{array} & \left(\begin{array}{ccccc} 560 & 0 & 0 & 0 & 0\\ 0 & 560 & 459 & 217 & 20\\ 0 & 459 & 560 & 217 & 20\\ 0 & 217 & 217 & 560 & 20\\ 0 & 20 & 20 & 20 & 560 \end{array}\right) \end{array}.$$

**Figure 2:**
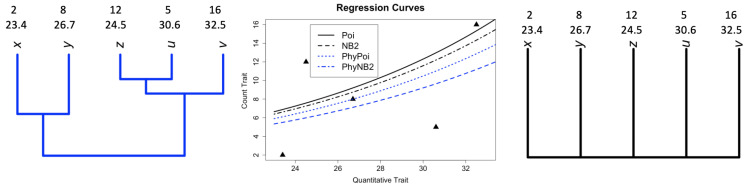
**Left** panel: phylogenetic tree and two traits. **Right** panel: star tree and two traits that assume independence. **Middle** panel: hypothetical regression curves with/without a tree. The regression curves under the GLM approach are reported in black (Poi for Poisson regression and NB2 for negative binomial regression); while the regression curves in blue (PhyPoi for phylogenetic Poisson regression model and PhyNB2 for phylogenetic negative binomial regression model) in blue incorporate the tree (C matrix) under the GEE technique [9] are sketched. The triangles (▲) are the 2D scatter observation for the response trait (count) vs. predictor trait (quantitative).

The conceptual regression curves shown in the upper-right panel of Figure 1 using two types of trees and a toy dataset with trait values Y=(2,8,12,5,16) for dependent count variable, and x=(23.4,26.7,24.5,30.6,32.5) for quantitative covariate trait variable are shown in Figure 2.

### 2.4. Leveraging GEEs for Regression Analysis of Phylogenetically Dependent Data

Trait evolution research [35], a crucial element in evolutionary biology, requires careful consideration of phylogenetic dependencies embedded within count data. A proven technique to handle these dependencies involves embedding a matrix C, extracted from the phylogenetic tree, in the regression model. This crucial integration accommodates species interrelationships, thereby facilitating precise interpretations. Our analysis primarily focuses on two types of regression models, namely Poisson and negative binomial regression, both members of the exponential family whose probability density function can be expressed in Equation (6) [36].

(6) $$f\left(y\right)=\exp\left(\frac{y\theta -b(\theta )}{a(\phi )}+c\left(y,\phi \right)\right).$$

GEE emerged as invaluable tools when applying these models. GEE prescribes a parameterization for θ, the distribution parameter of the exponential family, using a link function g(·) that associates the mean function μ and the variance function *V* of the response variable to the model’s linear predictors. Subsequently, the first two moments of *y* (μ and *V*), are represented through a series of functional relationships that encompass the parameters θ, μ, η, and $\beta =(\beta_{0},\beta_{1},\cdots ,\beta_{p})$ where η=g(μ)=Xβ where $X=[x_{0},x_{1},x_{2},\cdots ,x_{p}]$ is a design matrix of n×(p+1) consisting of $x_{0}={\left(1,1,\cdots ,1\right)}^{t}$ (the vector of 1s) and the covariates $x_{j}=\left(x_{1j},x_{2j},\cdots ,x_{nj}\right),j=1,2,\cdots ,p$ [9]. The final estimation equation for the regression parameter β is obtained by setting the derivative of the (p+1) estimating equations shown in Equation (7) to zero.

(7) $${\left[{\left\{\sum_{i=1}^{n}\left(\frac{y_{i}-\mu_{i}}{a\left(\phi \right)V\left(\mu_{i}\right)}\right){\left(\frac{\partial \mu }{\partial \eta }\right)}_{i}x_{ij}\right\}}_{j=0,1,2,\cdots ,p}\right]}_{(p+1)\times 1}={\left[0\right]}_{(p+1)\times 1}.$$

In the ensuing subsections, we delve deeper into the application of GEE in the domain of phylogenetic trait evolution analysis. We study it in two contexts: the widely acknowledged phylogenetic Poisson regression model and an emerging model, the phylogenetic negative binomial regression model. Given that these regression models are not extensively examined in the current literature, our efforts aim to illuminate their usage and implications, thereby contributing to a broader understanding of phylogenetic trait evolution. Of particular note is the incorporation of the C matrix into the GEE when solving to obtain the estimators (see Equation (9) for Poisson regression case and Equation (11) for negative binomial case). This integration is key to our models where the phylogenetic correlated and dependence among species are used, and the advantages it offers are explicitly discussed in Appendix A, where we lay out the more intricate mathematical details for comprehensive access and understanding. The detailed mathematical formulations of these models are provided in the Appendix A.4, with a specific mention in Appendix A.4.1 and Appendix A.4.2.

#### 2.4.1. Utilizing GEE in Phylogenetic Poisson Regression

Within the domain of evolutionary biology, GEE have become an indispensable tool for scrutinizing count data with inherent correlation structures. This correlation could either be explicitly defined or need estimation. GEE can work with various correlation structures, including independence, exchangeable, autoregressive order 1, and unstructured, as discussed in [15].

A pioneering application of GEE in comparative biology was presented by [9], where the correlation structure is derived from a phylogenetic tree, thereby accounting for the evolutionary interrelations between species. This framework significantly broadens the ability to analyze comparative data, particularly within the Poisson regression model context.

Given a group of *n* species associated with a trait vector $Y=(y_{1},y_{2},\cdots ,y_{n})$. Consider a count response variable $y_{i}$ for the *i*th observation with an associated mean rate $\lambda_{i}$. The density function for this variable follows a Poisson distribution and can be represented in an exponential form through a simple logarithmic transformation ($\theta_{i}=g\left(\lambda_{i}\right)=\log\left(\lambda_{i}\right)$). Within the GEE framework, the first and second moments, $E[y_{i}]$ and $V[y_{i}]$, can be derived directly from the link function’s derivatives and its inverse Equation (8).

(8) $$\begin{array}{cc} \mu =E\left(y\right)=b^{\prime }\left(\theta \right)=\lambda , & \\ \sigma^{2}=V\left(y\right)=b^{\prime \prime }\left(\theta \right)a\left(\phi \right)=\lambda . \end{array}$$

This approach enables a robust calculation of both the expected value and variance of the response variable, taking into account the phylogenetically structured correlation in the data.

GEE is used to estimate regression parameters in β, employing the chain rule to compute the derivative of the negative log-likelihood function. This process yields an expression involving the *i*th regression parameter’s partial derivative, which can be cast into matrix form, offering a comprehensive perspective on the regression estimates across all observations and parameters. The variance-covariance matrix was further refined [9] for use in phylogenetic comparative analyses, proposing as a combination of the phylogenetic correlation matrix C. The general estimating equation in Equation (7) can be written in matrix form shown in Equation (9).

(9) $$X_{(p+1)\times n}^{t}\lambda_{n\times 1}1_{1\times n}^{t}{\left(A_{n\times }^{1/2}C_{n\times n}A_{n\times }^{1/2}\right)}^{-1}\left(Y_{n\times 1}-\lambda_{n\times 1}\right)={\left[0\right]}_{(p+1)\times 1},$$

where $\lambda =(\lambda_{1},\cdots ,\lambda_{n})$ and $A=\mathrm{diag}(\exp\left(\sum_{j=0}^{p}\beta_{j}x_{ij}\right)),i=1,2,\cdots ,n$).

Given a set of response variables y and design matrix X, the regression parameters β can be estimated by solving this nonlinear equation system, providing an exhaustive characterization of trait data within their phylogenetic context (see Appendix A.3.1).

#### 2.4.2. Applying GEE in Negative Binomial Regression

In biological research, the GEE method is in a need of being utilized to perform negative binomial regression. This approach is primarily due to its ability to accommodate overdispersion commonly observed in biological data. It also facilitates adjustments for non-independence resulting from repeated measures, phylogenetic structure, or spatial and temporal autocorrelation, offering significant benefits for applications in evolutionary ecology, population biology, and comparative phylogenetics [37].

In this section, we explore the application of the GEE in negative binomial regression, emphasizing its use in phylogenetic comparative methods. The negative binomial distribution is characterized by parameters *r* and *p*, which correspond to the number of successes and the success probability in each trial, respectively.

To conduct a negative binomial regression using the GEE, we employ the canonical log-link function, linking the mean response to the linear predictors. This log-link function, in the context of negative binomial regression, is expressed in terms of *r* and the mean response μ (i.e., $\theta =\log\frac{\mu }{\mu +r}$). Implementing the GEE necessitates specifying the mean, link, and variance functions. In a negative binomial regression context, the mean function E[y] and the variance function can be written as in Equation (10)

(10) $$\begin{array}{ccc} \mu & =E(y)=r\exp(\theta )/(1-\exp(\theta )), & \\ \sigma^{2} & =V\left(y\right)=r\exp\left(\theta \right)/{\left(1-\exp\left(\theta \right)\right)}^{2}=\mu +\frac{\mu^{2}}{r}. \end{array}$$

To determine the regression estimates for $\beta_{i}$, we express the link function and the variance function in terms of the observed variables and $\beta_{i}$. Subsequently, we compute the partial derivative of $\mu_{i}$ with respect to $\eta_{i}$, which is crucial for solving the GEE in Equation (7).

From the foundational assumptions, we can derive estimating equations for the regression parameters β. These equations, also referred to as GEE and seen in Equation (7), serve as consistent estimators of β. Their expression in a matrix form, depicted in Equation (11), greatly facilitates solving the nonlinear system for β. In the development of the phylogenetic negative binomial regression, the GEE is transformed into a matrix form to encapsulate the phylogenetic correlation matrix, C. This matrix encodes the phylogenetic relationships among species. The process of integrating C into deriving the phylogenetic negative binomial regression can be represented by the matrix equation in Equation (11).

(11) $$X_{(p+1)\times n}^{t}\mu_{n\times 1}1_{1\times n}^{t}{\left(B_{n\times n}^{1/2}C_{n\times n}B_{n\times n}^{1/2}\right)}^{-1}\left(Y_{n\times 1}-\mu_{n\times 1}\right)={\left[0\right]}_{(p+1)\times 1},$$

where $\mu =(\mu_{1},\cdots ,\mu_{n})$ and $B=\mathrm{diag}\left(V\left(y_{i}|x_{i}\right)\right)=\mathrm{diag}\left(\mu_{i}\right)=\mathrm{diag}\left(r\exp\left(\sum_{j=0}^{p}x_{ij}\beta_{j}\right)/\left(1-\exp\left(\sum_{j=0}^{p}x_{ij}\beta_{j}\right)\right)\right)$. This matrix-based expression of the GEE facilitates solving the nonlinear system for β (see Appendix A.3.2).

The GEE offers a flexible and robust approach to modeling phylogenetic comparative data using negative binomial regression, especially in the presence of overdispersion. Effectively incorporating this into phylogenetic comparative methods can significantly advance our understanding of evolutionary patterns and processes. To test for the significance of the effect, we use the bootstrap technique [38] to generate the samples and re-estimate the parameters for constructing the confidence interval for the empirical analysis. The bootstrap means and the standard error for the regression parameter are reported.

## 3. Results

To assess the efficacy of our proposed method, we conducted a simulation focused on evaluating the parameter estimation of both regression models. Details regarding the simulation process can be found in Section 3.1. Furthermore, the outcomes specific to the phylogenetic Poisson regression model and the phylogenetic negative binomial regression model are presented in Section 3.1.1 and Section 3.1.2, respectively.

### 3.1. Simulation

To evaluate the method, we performed a simulation to assess the two regression models in the aspect of parameter estimation. The simulation uses four taxa sizes: *n* = 16, 32, 64, 128 and 4 types of trees: coalescent tree, balanced tree, left tree, and star tree. One covariate is used for the assessment of the model and the true parameter for $\left(\beta_{0},\beta_{1}\right)$ is set to (3,5). Subsequently, the parameters for simulating responses are computed using the mean function and variance function for the Poisson distribution (as shown in Equation (8)), and the Negative Binomial distribution (as shown in Equation (10)), respectively. The simulation uses 1000 replicates.

Simulate discrete trait: The ordsamplep.poi function we created initiates the generation of simulated data for a phylogenetic Poisson regression model. It produces values from a multivariate normal distribution with zero mean and covariance matrix C derived from the phylogenetic tree. These values are then transformed into Poisson-distributed variables using the qpois function, aligning with a Poisson distribution for a particular mean function λ parameter. Consequently, the simulated data mimics count traits with phylogenetic correlation, well-suited for phylogenetic Poisson regression analysis.

Similarly, the ordsamplep.nb2 function we created, backed by the MASS library [39], generates simulated data for the phylogenetic negative binomial regression model. It begins by creating random multivariate normal distribution values, consistent with the variance-covariance matrix C of the phylogenetic tree. These values are then transformed into negative binomially distributed variables using the qnbinom function with a negative binomial distribution for a particular mean function μ parameter. As a result, the simulated data manifests count traits with phylogenetic dependencies, providing an ideal testing ground for the phylogenetic negative binomial regression model.

When scaling the tree, each branch is assigned a length of less than 1. This can result in zero counts being generated due to the short branch lengths when using count random generators such as a Poisson or negative binomial. Hence, it is imperative to give careful consideration to tree lengths, especially when assessing discrete character changes. Trees of shorter lengths tend to show minimal variation, often exhibiting just 0, 1, or 2 changes from their root to their tip. Hence, expanding these trees by adding more tips might not yield much additional information. Conversely, for elongated trees that average around 15 changes, the varied branches could be more informative, potentially leading to more refined estimates. Instead of merely normalizing tree height, there is merit in exploring the dynamics of taller trees.

Simulate quantitative covariate trait: the predictive trait can be assumed to follow a Brownian motion with root value μ=3 estimated from the Brownian motion model [40] with rate parameter σ=1. This can be directly applied to the multivariate normal distribution $x∼N_{n}\left(\mu 1,\sigma^{2}C\right)$ as the joint distribution for each Brownian motion random variable is again a normal distribution [33,41]. For non-normal distributed trait, one can considere to simulate the covariate X from the exponential distribution with a known rate parameter.

#### 3.1.1. Phylogenetic Poisson Regression

The response data Y are simulated using the quantile function of the Poisson distribution with the specified mean $\lambda =\exp(\beta_{0}+\beta_{1}x)$ and the covariate x simulated by the multivariate normal distribution with mean 0 and covariance C. Then, the phylogenetic Poisson regression model is fitted to the samples. For each taxon and tree type case, 1000 samples are simulated and the mean estimates and standard deviation for the regression parameters are reported in Table 2.

In Table 2, parameter estimates for a phylogenetic Poisson regression model under four types of tree (coalescent, balanced, left, star) and four taxa sizes (16,32,64,128) are presented. Specifically, it reports the mean and standard deviation (in parentheses) of the estimates for the parameters $\beta_{0}$ and $\beta_{1}$. Furthermore, the means of the parameter estimates seem to be fairly consistent across the various taxa sizes for each tree type. This indicates the robustness of these estimates to the size of taxa considered in the model.

**Figure 3:**
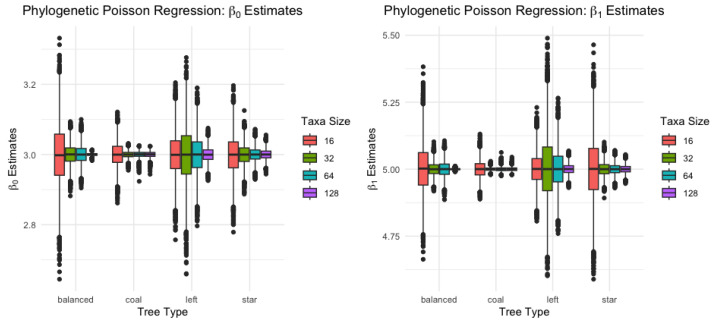
Phylogenetic Poisson regression $\beta_{0}$ and $\beta_{1}$ estimates.

One important observation from the table is the trend of the standard deviations across different taxa sizes, as also shown in Figure 3. For each tree type and parameter ($\beta_{0}$ and $\beta_{1}$), the standard deviation appears to decrease as the taxa size increases from 16 to 128. This suggests that the precision of the parameter estimates improves with increasing taxa size, which is consistent with the idea that larger sample sizes generally provide more precise estimates in statistical analyses. In other words, the estimates for $\beta_{0}$ and $\beta_{1}$ become more reliable and less variable with the increase in taxa size.

#### 3.1.2. Negative Binomial Regression

**Figure 4:**
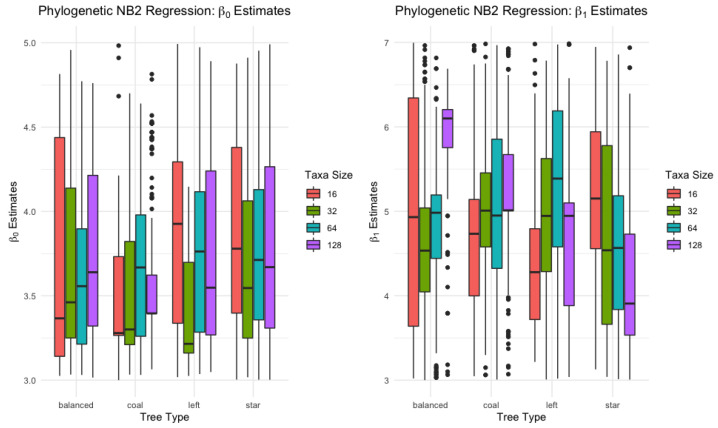
Phylogenetic negative binomial regression $\beta_{0}$ and $\beta_{1}$ estimates.

Given the covariate samples x, true parameters $\beta_{0}=3,\beta_{1}=5$ and *r* which is set to 10.68. The response data Y are simulated of the negative binomial distribution with specified mean $\mu =r\exp\left(\beta_{0}+\beta_{1}X\right)/(1-\exp\left(\beta_{0}+\beta_{1}x\right)$ with dispersion parameter 1/r. Then, the phylogenetic negative binomial regression model is fitted to the samples. For each taxon and tree type case, 1000 samples are simulated, and the mean estimates and standard deviation for the regression parameters are reported in Table 3 and Figure 4.

The parameter estimation results as shown in Table 3 and Figure 4 give valuable insights into the behavior of phylogenetic negative binomial regression across different tree types and taxon sizes.

From the Table 3, it becomes clear that as the taxa size increases, the mean estimates for the intercept ($\beta_{0}$) tend to converge more closely to their true values. Meanwhile, the mean estimates for the slope ($\beta_{1}$) are close to the true value, albeit with a relatively larger standard deviation. This observation reinforces that the phylogenetic negative binomial regression model is performing within expectations, demonstrating its capability to furnish relatively precise parameter estimates across varied conditions. Yet, a deeper exploration into the nuances of parameter estimation within this model reveals challenges in identifying a consistent overarching trend. Some taxa sizes exhibit pronounced variability, marked by significant standard deviations, complicating any straightforward trend interpretation. The quest for consistency across different tree types also proves elusive. This deviation is in sharp contrast to the more discernible patterns typically observed in the phylogenetic Poisson regression model. Such disparities underscore the intricate challenges associated with the phylogenetic negative binomial regression, especially when juxtaposed against other regression frameworks.

One explanation for these larger variations can be found in the nature of the estimation process itself. As mentioned in the text, the estimation of these parameters includes the solving of nonlinear equations (see Equation (11)). Such equations, especially when applied to complex biological data such as phylogenetic trees, can lead to a wide range of solutions. This might explain the relatively large standard deviations observed in these results. It is also worth mentioning that while some variability in the estimates is expected and indeed necessary for the model to adapt to different data structures, overly large variances might compromise the precision of the model. Therefore, this is a point that might warrant further investigation and potential refinements to the model or the estimation process.

As shown in Table 3 and Figure 4, the high variances could impact the precision of the model. These variances could be a result of the complexity involved in solving nonlinear equations, especially in complex biological data such as phylogenetic trees. Strategies to manage such issues could include employing better algorithms, as will be discussed later, to enhance the solution-finding process. Additionally, lowering the tolerance could help minimize the divergence in results.

By comparing the two models via Table 2 and Table 3. Upon comparing the phylogenetic Poisson regression and phylogenetic negative binomial regression models, one notices key differences. The phylogenetic Poisson regression model shows consistent parameter estimates for different taxa sizes, with values for $\beta_{0}$ and $\beta_{1}$ closely clustering around the true values of 3 and 5, respectively, across various tree types. This consistency is accompanied by a remarkably small standard deviation, suggesting a high degree of precision. In contrast, the phylogenetic negative binomial regression model displays more variability in its estimates. Although the values of $\beta_{0}$ and $\beta_{1}$ are in close proximity to the true values, they diverge more than the phylogenetic Poisson regression model’s estimates. Additionally, the larger standard deviations point towards greater uncertainty. Despite the higher variability, phylogenetic negative binomial regression could be more suitable under less predictable conditions, while phylogenetic Poisson regression provides stable estimates, proving reliable under steady scenarios.

### 3.2. Empirical Analysis

Building upon our simulation results, we proceeded to apply our proposed models to real-world empirical datasets. These results served to contextualize and validate our simulated observations, enabling us to examine the models’ efficacy in real-life scenarios. The patterns of variability noted in the simulations across tree types and taxa sizes were echoed in the empirical studies, reinforcing our understanding of these dynamics. The use of the phylogenetic negative binomial regression model on the lizard and mammalian datasets also emphasized the model’s applicability to count variables in a real biological context. Thus, these empirical analyses provide tangible insights that complement and substantiate our simulation findings.

In our empirical analysis, we currently make use of two different datasets, as outlined in Table 1. The first dataset refers to lizards, with a specific focus on egg count (a count variable) [21]. The second dataset is derived from mammalian data, where the variable of interest is the size of the litter, which refers to the simultaneous live birth of multiple offspring of a single mother [22].

The efficacy of the phylogenetic negative binomial regression model is tested against these two datasets. In Section 3.2.1, we apply this model to the lizard dataset to examine egg count in relation to body mass [21]. For the mammalian dataset, detailed in Section 3.3, we use this model to investigate litter size in response to factors such as number of teats, litter size, longevity, and body mass [22]. These empirical assessments serve to underscore the utility of the phylogenetic negative binomial regression model in the study of count variables.

#### 3.2.1. Lizard’s Egg-Laying Count

In various species observed in nature, there appears to be an inverse relationship between egg mass and the number of eggs laid per incubation. For instance, despite having a similar body size to chickens, the kiwi bird produces only one egg, while chickens lay multiple eggs. In our research, we have employed data that were previously collected and studied by [21]. This data primarily focus on the body size, represented as Snout–Vent Length (SVL), of the lizard species *S. undulatus*. Covariates such as age at maturity, egg mass, clutch size, and total eggs were incorporated in the regression analysis, with the response variable being the number of litters.

To enhance the reproducibility of our methodology, we have thoroughly detailed our data pre-processing steps. Initially, the raw data from [21] was collected and compiled in Table A1, found in Appendix A.2.1. This table illustrates the mean values of life history count variables for all Sceloporus populations, with the sources for the life-history data and mtDNA specified in the final two columns [42].

**Figure 5:**
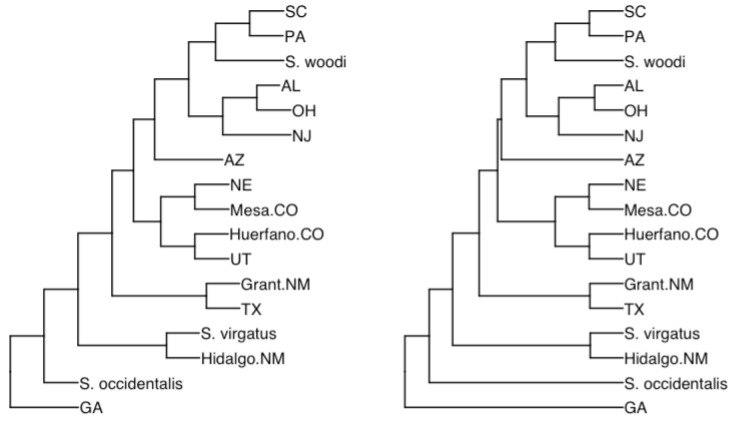
The comparative analysis is rooted in a phylogenetic tree of Sceloporus undulatus populations. **Left**: the original tree adapted from a comprehensive tree with branch lengths described by [42] and modified to exclude any populations lacking life-history data. The annotations on the revised tree highlight the state and county of mtDNA sample collection. **Right**: to fit our dataset, this tree is fine-tuned and subsequently transformed it into an ultrametric format using ape::chronopl [43]. The visualization of the tree was achieved with ggtree [44].

We then employed this dataset in our regression analyses, correlating the aforementioned covariates with the number of litters. It is worth mentioning that the phylogenetic tree of the lizard is also based on the study by [21] and is visually represented in Figure 5. The entire process ensures a comprehensive and replicable approach to analyzing the data, thus ensuring the robustness of our findings.

The regression estimates for the model are shown in Table 4.

Both the Poisson regression coefficient and the negative binomial regression coefficient can be interpreted as follows: for a one-unit change in the predictor variable, the difference in the logs of expected counts of the response variable is expected to change by the respective regression coefficient, given the other predictor variables in the model are held constant.

- In the negative binomial regression (glm.nb), the Egg Mass (EM) coefficient (${\hat{\beta }}_{1}$) is −1.188. In practical terms, an increase in Egg Mass by one unit results in a decrease in the log of expected counts of Eggs Per Year (EPY) by 1.188 unit. This model, with a standard deviation of 0.735, confirms the inverse association between egg size and the number of eggs laid per year.
- The Poisson regression regression (glm.poi) exhibits an EM coefficient (${\hat{\beta }}_{1}$) of −3.302. Meaning, an increase in EM by one unit leads to a decrease in the log of expected counts of EPY by 3.302 unit. With a standard deviation of 0.566, this model reveals a more pronounced inverse relationship between egg size and annual egg production compared to the negative binomial models.
- In the phylogenetic negative binomial regression (phygee.nb), the coefficient of EM (${\hat{\beta }}_{1}$) is −1.258. This indicates that an increase in EM by one unit results in a 1.258 unit reduction in the log of expected EPY counts. With a standard deviation of 1.065, this phylogenetic model indicates a slightly stronger inverse correlation between egg size and number laid per year than the glm.nb model.
- The phylogenetic Poisson regression via GEEs (phygee.poi) present an EM coefficient (${\hat{\beta }}_{1}$) of −2.831. This suggests that for every increase in EM of one unit, the log of expected EPY counts decreases by 2.831. The model has a standard deviation of 0.569. Although the phylogenetic model demonstrates a less pronounced effect of egg mass on yearly egg production than the non-phylogenetic Poisson model, it still exhibits a stronger correlation than the negative binomial models.

The comparative analysis of these four models provides some valuable insights. It is noteworthy that the negative binomial models (both general and phylogenetic) show a consistent negative relationship between egg size and annual egg production, albeit with slightly smaller effect sizes. This aligns with existing studies, which also suggest this inverse relationship. However, our work enhances the understanding of this relationship by employing both GLMs and generalized estimation equations, which capture and consider the evolutionary relationship between species.

**Figure 6:**
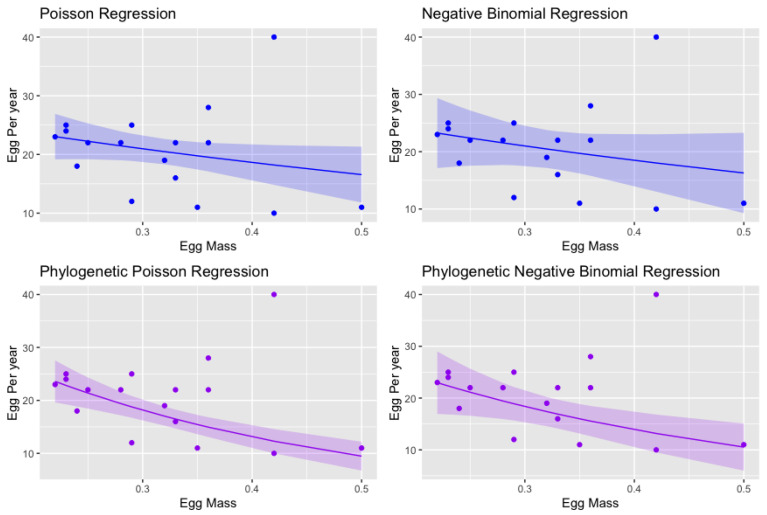
Regression curves for count data: Poisson (**upper left**), negative binomial (**upper right**), phylogenetic Poisson (**lower left**), and phylogenetic negative binomial (**lower right**). Predicted values, ${\hat{y}}_{i}$, form the curves. For Poisson-type regressions, ${\hat{y}}_{i}$ equals $\exp({\hat{\beta }}_{0}x_{i0}+{\hat{\beta }}_{1}x_{i1})$, and for negative binomial-type regression, it equals $(\hat{r}\exp\left({\hat{\beta }}_{0}x_{i0}+{\hat{\beta }}_{1}x_{i1}\right)/\left(1-\exp\left({\hat{\beta }}_{0}x_{i0}+{\hat{\beta }}_{1}x_{i1}\right)\right)$. The fitted.values function retrieves these values for GLM models. For phylogenetic models, the predict function follows the usage of compar.gee [31] for phylognetic poisson regression and compar.gee.nb2 created for phylogenetic negative binomial regression, retrieving the estimates.

In comparison, the Poisson models (both non-phylogenetic and phylogenetic) indicate a more pronounced inverse relationship between egg size and annual egg production, which extends the findings of previous research. These results suggest that the use of different statistical models can reveal nuanced details about biological relationships that would not be as evident with a single model. The regression curves are presented in Figure 6.

In summary, the regression models in Table 4 suggest a consistent trend across both negative binomial and Poisson regressions, and their respective phylogenetic versions. All point towards the same biological interpretation: larger egg sizes are associated with fewer eggs being laid per year, with this effect being somewhat stronger in the Poisson models. As illustrated in Figure 6, the negative binomial regression exhibits greater variation and broader confidence intervals than the Poisson regression, whether in phylogenetic or standard contexts. It is worth noting that various genetic and environmental factors can influence egg size in lizards, including the lineage, ambient temperature, and overall health of the animal. A critical observation is the apparent trade-off between egg size and the number of eggs produced annually, potentially representing an adaptive response to optimize offspring survival. Larger eggs might yield stronger, more resilient offspring, but at the cost of reduced egg quantity. This trade-off carries implications for reproductive strategies, population dynamics, and the broader evolutionary course of different lizard species. Understanding this phenomenon further would yield important insights into lizard life history strategies and their responses to environmental changes.

### 3.3. Litter Size in Mammal

In mammals, there is a general pattern where the maximum litter size is often constrained by the number of teats, and typically, the average litter size is about half the number of teats. This trend, however, can vary across different species [45]. For instance, the naked mole-rat (*Heterocephalus glaber*) presents an interesting deviation. It has approximately 12 nipples, but its average litter size is about 11 pups, significantly higher than the typical half. Moreover, the litter size can range from 3 to 12 pups and can even reach as high as 28 in some instances [46].

**Figure 7:**
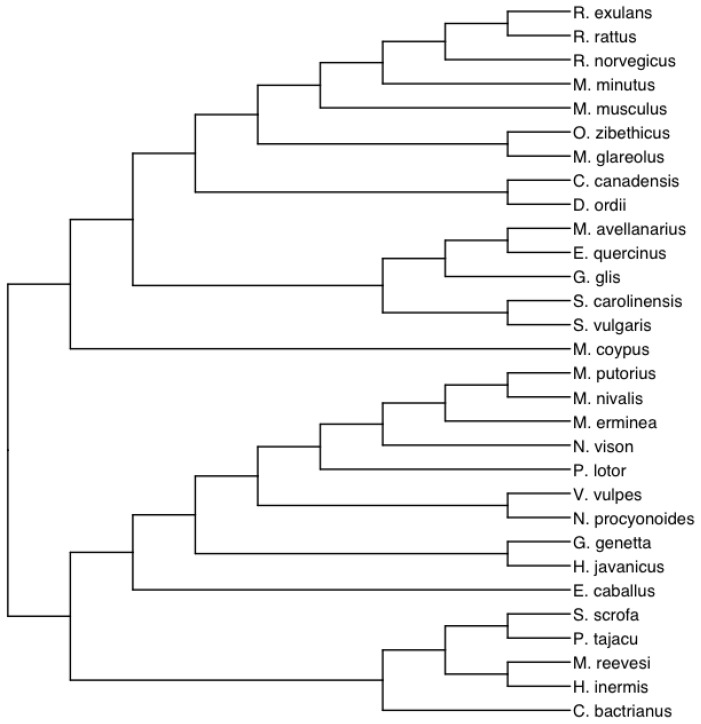
This figure presents a mammalian phylogenetic tree obtained from Phylotastic [48], analogous to [49]. It features 30 species with full data across all four study traits.

The need for a comprehensive understanding inspired us to devise a new methodology. Our study incorporates the collection of data pertaining to mammal litter sizes and other traits, such as body mass, gestation length, weaning age, height, and other relevant measurements, as detailed in [22]. The trait data depicted in Table A2 was obtained from [47] (see Appendix A.2.2). We further integrated the mammalian phylogenetic tree, as shown in Figure 7, derived from Phylotastic [48] in a manner similar to [49]. The featured phylogeny encompasses 30 species with complete datasets across all four traits under consideration.

Having discussed the collection and integration of the data, it is crucial to expound on how this gathered data is utilized. This brings us to the application of statistical models, which provide the framework for interpreting the information and yielding insightful findings. Under the assumption that the observations are independently distributed, parameter estimation falls within the purview of the GLM. Progressing to phylogenetic negative binomial regression analysis, initial estimates of parameters are computed using the R package glm with the Poisson family. This step solidifies the foundation for our subsequent analysis, ensuring that our data are primed for generating robust conclusions.

The regression estimates for the model are shown in Table 5.

- In the negative binomial regression (glm.nb), biological factors impact the expected log count of Litter Size per Year (LY). A one-unit increase in Litter Mean Body Size (LS) or Offspring Value (OV) reduces the log count of LY by −0.135 and −1.409 unit respectively, all else being equal. Longevity (LG) also has a smaller, negative impact, with a −0.047 decrease per unit increase. Contrastingly, a unit increase in Spread (SP) increases the LY log count by 0.47 unit.
- For the phylogenetic negative binomial regression (phygee.nb), the same biological factors show slightly altered impacts but maintain their directions. The log count of LY decreases by −0.143, −1.479, and −0.048 unit with each unit increase in LS, OV, and LG, respectively. However, a unit rise in SP increases the LY log count by 0.478 unit.
- In the Poisson regression (glm.poi), each unit increase in LS, OV, and LG reduces the log count of LY by −0.235, −2.572, and −0.058 unit, respectively. Conversely, a unit rise in SP increases the log count of LY by 0.515 unit.
- In the phylogenetic Poisson regression (phygee.poi), each unit increase in LS, OV, and LG leads to a decrease in the log count of LY by −0.231, −2.621, and −0.059 unit, respectively. In contrast, a unit rise in SP increases the LY log count by 0.521 unit.

In summary, across all models, an increase in each of LS, OV, and LG while holding all other predictors in the model is associated with a decrease in the expected log count of LY, while an increase in SP is associated with an increase in the expected log count of LY. However, the magnitude of these impacts varies between the models. While the Poisson models generally estimate larger effects than the negative binomial models, the negative binomial models accounts for larger variation than the Poisson models. In addition, the phylogenetic models estimate slightly different impacts compared to their non-phylogenetic counterparts.

## 4. Discussion and Conclusions

### 4.1. Improving Traditional Regression Models

The usage of traditional regression models such as the GLMs may not always yield accurate results due to their assumptions of data independence, which does not hold true for trait data from related species. To overcome this, we have proposed a novel phylogenetic negative binomial regression model that takes into account the inherent dependence arising from shared ancestry. Estimations within this model are carried out using the GEE framework, ensuring a comprehensive analysis. Through rigorous bootstrapping simulations, we assess the model’s effectiveness and demonstrate its practical application on empirical data obtained from a field study. Thus, our proposed model provides a more precise tool for analyzing count-dependent variables in related species, ultimately contributing to a more nuanced understanding of these relationships. Our model overcomes limitations inherent in traditional regression models, considering the inherent dependencies arising from shared ancestry. Hence, this model provides a robust and innovative tool for scientists conducting research on related species, thereby enhancing the quality and precision of findings in evolutionary biology and related fields.

### 4.2. Navigating Technical Challenges and Limitations

While our phylogenetic negative binomial regression model improves on traditional models such as GLM, it has limitations. Specifically, parameter estimation within our model requires advanced techniques to ensure rapid and convergent estimates. Moreover, our current model does not account for within-species variation, a scenario that warrants further exploration in future work. When accessing the robustness of our proposed phylogenetic negative binomial regression model, we estimated parameters using methods designed to solve systems of nonlinear equations. This was performed within a simulation under a bootstrapping approach. However, due to the high volume of repeated values in the simulated data, we occasionally encountered issues such as errors in singular value decomposition (SVD) when computing the inverse in the GEEs inverse in Equation (11). It is worth noting that these nonconvergent results reflect the inherent intricacies of the model and the potential limitations of the estimation method under specific conditions. In some cases, we had to reduce the tolerance during the decomposition of the equations to ensure a feasible estimate. However, this adjustment can occasionally lead to numerical instability, demonstrating the challenges inherent in balancing precision and computational stability in these complex models.

### 4.3. Exploring the Impact of Advanced Stochastic Processes in Phylogenetic Trait Evolution

The model can be expanded to accommodate additional evolutionary phenomena embbeded in the covariate trait x. For instance, Paradis [9] assumed no elements from the correlation matrix C needed estimation, as they were directly derived from the given tree. However, expanding this assumption to include other processes with parameters within C could provide a more comprehensive understanding of evolution. This could involve the Ornstein–Ulenbeck process model with a force parameter α [50], Pagel’s λ model with a scale parameter λ [51], or the early burst model to allow for adaptive trait evolution [52].

The recent advancements in the field of stochastic processes have been substantial, particularly in the context of Ornstein–Uhlenbeck (OU) and fractional Brownian motion models. These models provide a comprehensive framework for analyzing trait evolution across phylogenetic trees, extending beyond the traditional scope of the Brownian motion stochastic process and accommodating greater complexity and flexibility [53,54]. Currently, models that incorporate random diffusivity have been explored [55,56,57]. In particular, the work of Wang et al. (2020) discusses residual non-ergodicity below the correlation time in the context of a fractional Brownian motion with random diffusivity [55]. Furthermore, they examined anomalous diffusion and non-ergodicity within heterogeneous diffusion processes using fractional Gaussian noise [58]. Meanwhile, models for scaled Brownian motion with random diffusivity were proposed by Dos Santos and Junior (2021) and Miyaguchi (2022) [56,57].

Building on these developments, the complex behaviors of these models have been the focus of more recent investigations. Hidalgo-Soria, Barkai, and Burov (2021) delved into the cusp of the non-Gaussian density of particles within a diffusing diffusivity model [59]. Additionally, Dos Santos, Menon Jr. and Cius (2022) utilized a superstatistical approach to explore the anomalous exponent for scaled Brownian motion [60]. These advances provide intriguing directions for future research. The influence of these more generalized stochastic processes on the results of our current study is an area of interest for further exploration. However, an in-depth examination of these models is beyond the scope of our current research. The implications of population size on the parameters of the underlying stochastic process, specifically the diffusion coefficient, are crucial, especially in large populations. Although these fluctuations might not drastically affect the results of empirical analyses, they could play a significant role in simulation studies. Our current study acknowledges the importance of these issues and advocates for their consideration in future research, further enriching the understanding of phylogenetic trait evolution.

### 4.4. Towards Multivariate Count Models

Further directions can be taken to build upon the present work. Initially, the focus can shift toward multivariate count models. While a univariate analysis offers a comprehensive understanding of single-variate distributions, multivariate analysis provides an understanding of interrelationships between multiple variables. In particular, it could be valuable to extend our efforts to multivariate Poisson or Negative Binomial distributions. A multivariate framework would permit the identification and examination of correlations between components. The geepack package’s mmmgee function, as documented by [61], could facilitate this investigation.

### 4.5. Bayesian Approaches and Algorithmic Considerations

From a Bayesian viewpoint, there are also opportunities for further exploration. For example, we could expand on regression methods related to within-subject variability, as illustrated in the species examined by [14]. Another extension could involve adapting the multivariate Poisson log-normal model (PLN) proposed by [62]. This model connects p-dimensional observation vectors $Y_{j}$ with Gaussian latent variables $Z_{j}$. Under PLN, these latent variables follow a normal distribution with a specified mean and covariance, while the observations adhere to an exponential distribution given these latent variables. The integration of phylogenetic tree structures within this model could allow for a more comprehensive analysis.

Looking ahead, we recognize the need to consider alternative algorithms that can better handle these challenging scenarios. In particular, the tree pruning algorithm [63] might offer an innovative way to alleviate these problems when applying the phylogenetic regression model to count dependent variables. Tree pruning could provide a way to simplify the phylogenetic tree, thus reducing the computational burden and improving the stability of the model estimation process. This approach serves as an example of how future research could continue to refine these models, enhancing their robustness and accuracy in the analysis of data that exhibit dependencies due to shared ancestry.

## 5. Conclusions

In addressing the analytical challenges associated with dependent variables in related species, we have developed a phylogenetic negative binomial regression model that effectively addresses the analytical challenges associated with count-dependent variables in phylogenetically related species. This model, utilizing the GEE framework, not only rectifies the limitations of conventional GLMs but also enhances the quality and precision of findings in evolutionary biology. Importantly, this innovation opens up new avenues for future research, particularly in refining these models and exploring more advanced algorithms. These future directions are essential to better handle complex data dependencies and improve the robustness and accuracy of analyses.

## Figures and Tables

**Table 1:** Statistical summary and regression under GLM for lizard datasets [21] and mammal [22] datasets. The taxon size *n*, the mean, and the variance for the response variable $Y=(y_{1},y_{2},\cdots ,y_{n})$ where each $y_{i}$ is of count value, the corrected Akaike Information Criterion (AICc) (see Equation (3)) and Akaike weight (*w*) (see Equation (4)) for each model are reported. NB2 is the abbreviation of the phylogenetic negative binomial model.

Lizard Data		Mammal Data	
**Statistics**	**Value**	**Statistics**	**Value**
taxon size	17	taxon size	74
mean (Y)	20.82	mean (Y)	1.99
var (Y)	55.03	var (Y)	2.37
Poisson AICc	116.81	Poisson AICc	228.84
NB2 AICc	116.43	NB2 AICc	229.52
Poisson *w*	0.45	Poisson *w*	0.58
NB2 *w*	0.55	NB2 *w*	0.42

**Table 2:** The parameter estimates for the phylogenetic Poisson regression. Mean values along with standard deviations are provided for four types of trees across four different taxa sizes.

	Balanced		Coalescent		Left		Star	
	$\beta_{0}$	$\beta_{1}$	$\beta_{0}$	$\beta_{1}$	$\beta_{0}$	$\beta_{1}$	$\beta_{0}$	$\beta_{1}$
16	2.998 (0.086)	5.002 (0.088)	3 (0.034)	5 (0.031)	2.999 (0.059)	5.001 (0.057)	2.999 (0.055)	5 (0.114)
32	3 (0.028)	5 (0.024)	3 (0.009)	5 (0.006)	2.998 (0.082)	5.003 (0.121)	3 (0.029)	5 (0.025)
64	3 (0.025)	5 (0.028)	3 (0.009)	5 (0.008)	3 (0.054)	5.001 (0.071)	3 (0.019)	5 (0.019)
128	3 (0.004)	5 (0.003)	3 (0.008)	5 (0.007)	3 (0.02)	5 (0.019)	3 (0.014)	5 (0.015)

**Table 3:** Parameter estimates for phylogenetic negative binomial regression. The mean and standard deviation under 4 types of tree and 4 taxa size are reported.

	Balanced		Coalescent		Left		Star	
	$\beta_{0}$	$\beta_{1}$	$\beta_{0}$	$\beta_{1}$	$\beta_{0}$	$\beta_{1}$	$\beta_{0}$	$\beta_{1}$
16	1.651 (0.673)	5.029 (1.343)	2.113 (0.864)	4.693 (0.917)	1.878 (0.913)	4.425 (0.929)	2.714 (0.951)	5.226 (1.078)
32	2.029 (0.823)	4.613 (0.924)	2.14 (0.826)	4.97 (0.856)	1.543 (0.548)	4.917 (1.035)	2.705 (0.891)	4.699 (1.117)
64	2.312 (0.831)	4.82 (0.832)	2.031 (0.779)	4.997 (1.008)	1.71 (0.686)	5.255 (1.087)	2.991 (0.899)	4.556 (0.918)
128	2.856 (0.91)	5.856 (0.677)	2.866 (0.802)	5.174 (0.741)	1.79 (0.777)	4.808 (1.052)	3.143 (0.889)	4.21 (1.02)

**Table 4:** The lizard dataset, sourced from [21], examines the relationship between eggs per year (EPY) and egg mass (EM). It employs bootstrapped estimates and standard deviations, following [38]. Regression methods under a GLM frameworks: glm.nb for the negative binomial model and glm.poi for the Poisson regression model; and their phylogenetic versions: phygee.nb for the phylogenetic negative binomial model and phygee.poi for the phylogenetic Poisson model.

	glm.nb	glm.poi	phygee.nb	phygee.poi
Intercept (${\hat{\beta }}_{0}$)	3.397 (0.237)	3.88 (0.172)	3.411 (0.344)	3.753 (0.176)
EM (${\hat{\beta }}_{1}$)	−1.188 (0.735)	−3.302 (0.566)	−1.258 (1.065)	−2.831 (0.569)

**Table 5:** The mammal dataset, inclusive of bootstrapping estimates and standard deviations, evaluates litter size per year (LY) in relation to litter mean body size (LS), offspring value (OV), longevity (LG), and spread (SP).

	glm.nb	phygee.nb	glm.poi	phygee.poi
Intercept (${\hat{\beta }}_{0}$)	2.13 (0.414)	2.153 (0.414)	2.497 (0.289)	2.492 (0.309)
LS (${\hat{\beta }}_{1}$)	−0.135 (0.105)	−0.143 (0.102)	−0.235 (0.105)	−0.231 (0.106)
OV (${\hat{\beta }}_{2}$)	−1.409 (1.458)	−1.479 (1.616)	−2.572 (0.815)	−2.621 (1.041)
LG (${\hat{\beta }}_{3}$)	−0.047 (0.014)	−0.048 (0.015)	−0.058 (0.011)	−0.059 (0.012)
SP (${\hat{\beta }}_{4}$)	0.47 (0.24)	0.478 (0.223)	0.515 (0.181)	0.521 (0.19)

## Data Availability

Data can be accessed in Appendix A or by reaching out to the author.

## Appendix A. Scripts, Datasets, and Models

### Appendix A.1. Code Availability

The script developed for this project can be accessed at the following link https://tonyjhwueng.info/phypoinb2reg (accessed on 27 July 2023).

- Table 1: https://tonyjhwueng.info/phypoinb2reg/mammal_lizard.html (accessed 27 July 2023).
- Figure 1: https://tonyjhwueng.info/phypoinb2reg/illufigpropv2.html (accessed on 27 July 2023).
- Figure 2: https://tonyjhwueng.info/phypoinb2reg/schematicplot.html (accessed on 27 July 2023).
- Table 2, Figure 3: https://tonyjhwueng.info/phypoinb2reg/phypoisim.html (accessed on 27 July 2023).
- Table 3, Figure 4: https://tonyjhwueng.info/phypoinb2reg/phynb2simb0b1box.html (accessed on 27 July 2023).
- Figure 5: https://tonyjhwueng.info/phypoinb2reg/lizardtree2.html (accessed on 27 July 2023).
- Table 4: https://tonyjhwueng.info/phypoinb2reg/lizardyoboot2.html (accessed on 27 July 2023).
- Figure 6: https://tonyjhwueng.info/phypoinb2reg/lizardyoboot3plot.html (accessed on 27 July 2023).
- Figure 7, Table 5: https://tonyjhwueng.info/phypoinb2reg/mammalyoboot2.html (accessed on 27 July 2023).
- Table A1: https://tonyjhwueng.info/phypoinb2reg/lizardyo2.html (accessed on 27 July 2023).
- Table A2: https://tonyjhwueng.info/phypoinb2reg/mammalplot.html (accessed on 27 July 2023).

### Appendix A.2. Trait Dataset

#### Appendix A.2.1. Lizard Trait Set

**Table A1:** Lizard trait set in [21]. EPY: eggs per year, EM: egg mass (g). This table depicts the means of life history count variables for all *Sceloporus* populations. Data sources, including life-history and mtDNA, are referenced in the final two columns [42].

No.	Species	EPY	EM
1	*S. undulatus* (GA)	23	0.33
2	*S. undulatus* (OH)	24	0.35
3	*S. undulatus* (AL)	25	0.28
4	*S. undulatus* (NJ)	18	0.36
5	*S. undulatus* (PA)	22	0.42
6	*S. undulatus* (SC)	22	0.33
7	*S. woodi*	12	0.25
8	*S. undulatus* (AZ)	25	0.29
9	*S. undulatus* (UT)	19	0.36
10	*S. undulatus* (Huerfano.CO)	22	0.32
11	*S. undulatus* (Mesa.CO)	16	0.42
12	*S. undulatus* (NE)	11	0.23
13	*S. undulatus* (TX)	28	0.22
14	*S. undulatus* (Grant.NM)	22	0.29
15	*S. undulatus* (Hidalgo.NM)	40	0.24
16	*S. virgatus*	10	0.23
17	*S. occidentalis*	11	0.50

#### Appendix A.2.2. Litter Size

**Table A2:** This table presents traits for 30 species [22], including litters number (LY), body size (LS), offspring value (OV), longevity (LG), and successful spread. Data are from [47,64], and other sources, encompassing variables from [65].

	Species	LY	LS	OV	Spread	LG
1	*Sciurus carolinensis*	2	2.98	0.02	1	24.00
2	*Sciurus vulgaris*	2	4.50	0.04	1	14.80
3	*Dipodomys ordii*	2	2.95	0.05	0	9.90
4	*Micromys minutus*	3	4.92	0.08	0	5.00
5	*Ondatra zibethicus*	2	6.55	0.05	1	10.00
6	*Mus musculus*	5	5.54	0.04	1	6.00
7	*Rattus exulans*	4	3.70	0.39	1	1.12
8	*Rattus norvegicus*	4	8.99	0.08	1	3.80
9	*Rattus rattus*	4	5.88	0.07	1	4.20
10	*Glis glis*	1	5.17	0.09	0	12.00
11	*Muscardinus avellanarius*	2	4.30	0.14	0	6.00
12	*Eliomys quercinus*	1	4.99	0.25	0	5.50
13	*Castor canadensis*	1	3.60	0.05	1	23.40
14	*Myodes glareolus*	4	4.31	0.06	1	4.92
15	*Myocastor coypus*	3	5.34	0.03	1	12.00
16	*Vulpes vulpes*	1	4.59	0.05	1	21.30
17	*Nyctereutes procyonoides*	1	6.36	0.07	1	16.60
18	*Procyon lotor*	1	3.06	0.05	1	21.00
19	*Mustela erminea*	1	6.74	0.08	1	12.50
20	*Mustela nivalis*	1	5.07	0.10	1	10.00
21	*Mustela putorius*	1	8.48	0.08	1	14.00
22	*Neovison vison*	1	4.50	0.09	1	11.40
23	*Genetta genetta*	2	2.29	0.02	1	34.00
24	*Herpestes javanicus*	2	2.21	0.05	0	10.00
25	*Equus caballus*	1	1.00	0.02	1	62.00
26	*Sus scrofa*	2	4.52	0.03	1	27.00
27	*Pecari tajacu*	2	1.56	0.02	0	31.50
28	*Camelus bactrianus*	1	1.39	0.03	1	40.00
29	*Hydropotes inermis*	1	3.00	0.08	0	13.90
30	*Muntiacus reevesi*	2	0.98	0.03	1	23.20

### Appendix A.3. Count Regression for Independent Data

In Appendix A.3.1, we discuss the independent Poisson regression. The independent negative binomial regression is elucidated in Appendix A.3.2.

#### Appendix A.3.1. Independent Poisson Regression

In Poisson regression, we assume that the response variable $y_{i}$ of the *i*th species is a count random variable following a Poisson distribution Poi(λ), where $\lambda_{i}$ is the expected frequency given a period of time [15]. The likelihood function given the comparative data $\left(y_{1},y_{2},\cdots ,y_{n}\right)$ under the independent case is

(A1) $$L\left(\lambda |y_{1},y_{2},\cdots ,y_{n}\right)=\prod_{i=1}^{n}\frac{\exp\left(-\lambda \right)\lambda^{y_{i}}}{y_{i}!}.$$

Let $\beta ={\left(\beta_{0},\beta_{1},\cdots ,\beta_{p}\right)}^{t}$, and X be the n×(p+1) design matrix composed by the column vectors $1,x_{1},\cdots ,x_{p}$ where $1={\left(1,1,\cdots ,1\right)}^{t}$ is a vector of 1 s and $x_{j}=\left[x_{ij}\right],$i=1,2⋯,n,j=1,2,⋯,p. The log-link function enables us to model the expected value of $y_{i}$ as a linear combination of predictor variables. Consequently, the coefficient in Poisson regression represents the difference in the logarithm of expected counts per unit change in the corresponding predictor variable. By using the log link function on the mean, one has $\log\left(\lambda \right)=\log\left(E\left[y_{i}|x_{i0},x_{i1},x_{i2},\cdots ,x_{ip}\right]\right)=\sum_{j=0}^{p}\beta_{j}x_{ij}$, and hence

(A2) $$\lambda =\exp\left(\sum_{j=0}^{p}\beta_{j}x_{ij}\right).$$

Taking the negative log of *L* in Equation (A1) and use Equation (A2), the negative log-likelihood function *ℓ* is shown in Equation (A3),

(A3) $$\begin{array}{cc} \ell (\beta |y,X)= & \sum_{i=0}^{n}\left(-\lambda +y_{i}\log\left(\lambda \right)-\log\left(y_{i}!\right)\right)\\ = & \sum_{i=0}^{n}\left(-\exp\left(\sum_{j=0}^{p}\beta_{j}x_{ij}\right)+y_{i}\sum_{j=0}^{p}\beta_{j}x_{ij}-\log\left(y_{i}!\right)\right). \end{array}$$

To obtain a maximum likelihood estimate for parameters $\hat{\beta }$, take the partial derivative of Equation (A3) with respect to $\beta_{j}$ yields the Equation (A4)

(A4) $$\frac{\partial \ell }{\partial \beta_{j}}=\sum_{i=1}^{n}x_{ij}\left(y_{i}-\exp\left(\sum_{j=0}^{p}\beta_{j}x_{ij}\right)\right),j=0,1,\cdots ,p.$$

Then, the MLE estimate of parameters ${\hat{\beta }}_{i},i=1,2\cdots ,p$ can be obtained by solving the system of equations in Equation (A4) through the numerical procedure such as Newton’s method [17]. Readers can please refer to [66] for a more detailed description.

#### Appendix A.3.2. Independent Negative Binomial Regression

In negative binomial regression, we assume that the response variable $y_{i}$ of the *i*th species is a random count variable following negative binomial distribution NB(r,p) where r>0 is the number of success until the experiment is stopped, $y_{i}$ is the number of failures until the experiment is stopped and *p* is the probability of success in each experiment [67].

The likelihood function given the comparative data $y=(y_{1},y_{2},\cdots ,y_{n})$ under the independent case is

(A5) L(p,r|y)=∏i=1nyi+r−1yipr(1−p)yi,

where yi+r−1yi=Γ(r+yi)Γ(yi+1)Γ(r) and Γ is a gamma function [68,69].

Use the log link function $\log\frac{pr}{1-p}=\sum_{j=0}^{p}\beta_{j}x_{ij}$ for the mean, one has

(A6) $$p=\frac{1}{1+r\exp(-\sum_{j=0}^{p}\beta_{j}x_{ij})}.$$

Let $\mu_{i}=\exp\left(\sum_{j=0}^{p}\beta_{j}x_{ij}\right)/\left(1+\exp\left(\sum_{j=0}^{p}\beta_{j}x_{ij}\right)\right)$. Taking the negative log of *L* in Equation (A5) and use Equation (A6), the negative log-likelihood function *ℓ* is shown in Equation (A7),

(A7) ℓ(β|y,X)=∑i=1nlogyi+r−1yipr(1−p)yi=∑i=1nlogyi+r−1yi−(r+yi)log1+rμi+yilog(rμi).

To obtain the maximum likelihood estimate for parameters $\hat{\beta }$, taking the partial derivative to Equation (A7) with respect to $\beta_{j}$, namely, $\frac{\partial \ell }{\partial \beta_{j}},j=0,1,2\cdots ,p$ as well as taking the derivative to Equation (A7) with respect to *r*, namely, $\frac{\partial \ell }{\partial r}$.

(A8) $$\begin{array}{cc} \frac{\partial \ell }{\partial \beta_{j}} & =\sum_{i=1}^{n}\frac{x_{ij}\left(y_{i}-\mu_{i}\right)}{1+\mu_{i}/r},j=0,1,2,\cdots ,p;\\ \frac{\partial \ell }{\partial r} & =\sum_{i=1}^{n}\left[r^{2}\left(\log\left(1+\frac{\mu_{i}}{r}\right)-\sum_{j=0}^{y_{i}-1}\frac{1}{j+r}\right)+\frac{r^{2}\left(y_{i}-\mu_{i}\right)}{r+\mu_{i}}\right]. \end{array}$$

Then, the MLE parameters ${\hat{\beta }}_{j},j=0,1,\cdots ,p$ and $\hat{r}$ can be obtained by solving the system of equations in Equation (A8) through the numerical procedure such as Newton’s method [17]. Readers can please refer to [67,69] for a more detail description.

### Appendix A.4. Count Regression under GEE for Phylogenetic Dependent Data

The GEE is a strategy for parameter estimation in GLMs, accounting for potential correlation structures in outcomes, both known and unknown [15]. Traditional applications of GEE propose several correlation structures, including independence, exchangeable, autoregressive order 1, and unstructured. The authors of [31] extended GEE to comparative count-dependent data analysis. For regression of phylogenetically dependent count data, we introduce a transformation matrix C derived from the phylogenetic tree to account for species interdependencies. Our primary models of interest are the Poisson regression and the negative binomial regression. Both models belong to the exponential family, where the associated random variable *y* has its density function [36].

(A9) $$f\left(y\right)=\exp\left(\frac{y\theta -b(\theta )}{a(\phi )}+c\left(y,\phi \right)\right).$$

Using GEE, we initial parameterize θ through a monotonic link function g(μ)=θ. We denote $E\left(y\right)=b^{\prime }\left(\theta \left(\mu \right)\right)$ as the mean function and $V\left(\mu \right)=b^{\prime \prime }\left(\theta \left(\mu \right)\right)$ as the variance function which can be represented as differentials of b(θ), as indicated in Equation (A10)

(A10) $$\begin{array}{cc} E\left(y\right)=b^{\prime }\left(\theta \right)=\mu , & \\ V\left(y\right)=b^{\prime \prime }\left(\theta \right)a\left(\phi \right). \end{array}$$

Let $\eta_{j}=g\left(\mu_{j}\right)=X_{j}^{t}\beta$, given the interlinked relationships among the parameters, the partial derivative of *ℓ* with respect to β produces the estimation equation, guided by the chain rule expressed in Equation (A11).

(A11) $$\frac{\partial \ell }{\partial \beta }={\left[\left(\frac{\partial \ell }{\partial \theta }\right)\left(\frac{\partial \theta }{\partial \mu }\right)\left(\frac{\partial \mu }{\partial \eta }\right)\left(\frac{\partial \eta }{\partial \beta }\right)\right]}_{(p+1)\times 1}.$$

The estimation equation for the regression parameter β is achieved by setting the derivative of the log-likelihood, *ℓ*, to zero which yields to the final estimating equation in Equation (A12).

(A12) $${\left[{\left\{\sum_{i=1}^{n}\left(\frac{y_{i}-\mu_{i}}{a\left(\phi \right)V\left(\mu_{i}\right)}\right){\left(\frac{\partial \mu_{i}}{\partial \eta_{i}}\right)}_{i}x_{ij}\right\}}_{j=0,1,2,\cdots ,p}\right]}_{(p+1)\times 1}={\left[0\right]}_{(p+1)\times 1}.$$

The following two subsections present our description to the GEE method for phylogenetic trait evolution [9]. We explore both the phylogenetic Poisson regression model [9] and the novel phylogenetic negative binomial regression model.

Appendix A.4.1 details the GEE application in the context of phylogenetic Poisson regression, while Appendix A.4.2 delves into its application for phylogenetic negative binomial regression. To our best knowledge, these regression models are not comprehensively covered in the existing literature. Our objective is to bridge this gap by providing an in-depth study of these regression models.

#### Appendix A.4.1. GEE for Phylogenetic Poisson Regression

In the following, we provide description for GEE procedure for the phylogenetic Poisson regression model (the reader can also refer to [9]).

Since the Poisson distribution with probability mass function $p\left(y|\lambda \right)=\frac{e^{-\lambda }\lambda^{y}}{y!}$ belongs to the exponential family, refer to Equation (A9) one has

(A13) $$\begin{array}{cc} \theta & =\log\lambda ,\\ a(\phi ) & =1,\\ b(\theta ) & =\lambda ,\\ c(y,\theta ) & =-\log(y!). \end{array}$$

Then, the mean function and the variance function are shown in Equation (A14).

(A14) E(y)=V(y)=λ.

To apply the GEE for searching the regression estimate for $\beta_{j}$, since $\theta_{i}=\log\lambda_{i},\lambda_{i}=\exp\left(\sum_{j=0}^{p}x_{ij}\beta_{j}\right):=\exp\left(\eta_{i}\right)$ and $\frac{\partial \lambda_{i}}{\partial \beta_{j}}=\sum_{j=0}^{p}x_{ij}\lambda_{i},i=1,2,\cdots ,n$, the derivative of the negative log-likelihood function *ℓ* with respect to the parameter $\beta_{j}$ can be computed by the chain rule in Equation (A12) which yields the partial derivative of the *j*th regression parameter in Equation (A15).

(A15) $$\frac{\partial \ell }{\partial \beta_{j}}=\sum_{i=1}^{n}\left[\frac{y_{i}-\lambda_{i}}{\lambda_{i}}\lambda_{i}x_{ij}\right],j=0,1,2,\cdots ,p.$$

Let $\beta =(\beta_{0},\beta_{1},\cdots ,\beta_{p})$ and $\lambda =(\lambda_{1},\cdots ,\lambda_{n})$, one can deduce Equation (A15) in matrix form as

(A16) $${\left(\frac{\partial \lambda }{\partial \beta }\right)}_{(p+1)\times n}^{t}V_{n\times n}^{-1}{\left(y-\lambda \right)}_{n\times 1}^{t}.$$

In the phylogenetic comparative regression model context, Ref. [9] defined the variance-covariance between the observation with

(A17) $$V=\phi A^{1/2}CA^{1/2}$$

where ϕ is dispersion parameter, C is the phylogenetic correlation matrix with elements $c_{ij}$ of the shared branch length of taxa *i* and *j* and A is a diagonal matrix in Equation (A18).

(A18) $$A=\phi \cdot \mathrm{diag}\left(V\left(y_{i}|x_{i}\right)\right)=\phi \cdot \mathrm{diag}\left(\lambda \right)=\phi \cdot \mathrm{diag}\left(\exp\left(\sum_{j=0}^{p}x_{ij}\beta_{j}\right)\right).$$

Therefore, given the response $Y={\left(y_{1},y_{2},\cdots ,y_{n}\right)}^{t}$ and design matrix $X={\left(1,x_{1},x_{2},\cdots ,x_{p}\right)}^{t}$, by Equation (A12) the parameter estimate for β can be obtained by solving the nonlinear equation system of β.

(A19) $$X_{(p+1)\times n}^{t}\lambda_{n\times 1}1_{1\times n}^{t}{\left(A_{n\times n}^{1/2}C_{n\times n}A_{n\times n}^{1/2}\right)}^{-1}\left(Y_{n\times 1}-\lambda_{n\times 1}\right)={\left[0\right]}_{(p+1)\times 1}.$$

Given trait data $(x_{ij},y_{i}),i=1,\cdots ,n;j=1,2\cdots ,p$ and the tree associated C matrix, an estimate of $\beta_{j}$s are estimated through a numerical search by solving the system of a nonlinear equation. The parameter estimate for β embedded can be obtained by solving the system of the nonlinear equation of β.

#### Appendix A.4.2. GEE for Negative Binomial Regression

For a negative binomial random variable *y*, y∼NB(r,p) where the parameter *r* is the number of successes and *p* is the probability of success in each trial. The probability mass function is p(y|p,r)=y+r−1y(1−p)rpy=y+r−1yexp(ylogp+rlog(1−p)). The first moment is $E\left[y\right]=\mu =\frac{pr}{1-p}$ which shows that the *p* can be expressed in terms of μ and *r* as $p=\frac{\mu }{\mu +r}$.

Substitute *p* with μ/(μ+r) into the probability mass function p(y|p,r), we have

(A20) p(y|p,r)=y+r−1yexpylogμμ+r+rlogrμ+r=expylogμμ+r−rlog(μ+r)1+rlogr+logy+r−1y.

Compare Equation (A20) with Equation (A9) one has a(ϕ)=1, $\theta =\log\frac{\mu }{\mu +r}$ which implies μ=rexp(θ)/(1−exp(θ)). Now consider rlog(μ+r)=rlog(rexp(θ)/(1−exp(θ))+r)=rlog(r/(1−exp(θ)))=rlogr−rlog(1−exp(θ)), one has b(θ)=−rlog(1−exp(θ)), and c(y,ϕ)=2rlogr+logy+r−1y. Hence, we have

(A21) θ=logμμ+r,a(ϕ)=1,b(θ)=−rlog(1−exp(θ)),c(y,ϕ)=2rlogr+logy+r−1y.

The mean function E[y] and the variance function can be written $E\left[y\right]=b^{\prime }\left(\theta \right)=r\exp\left(\theta \right)/\left(1-\exp\left(\theta \right)\right)$, and the variance function V[y] is $V\left[y\right]=b^{\prime \prime }\left(\theta \right)a\left(\phi \right)=r\exp\left(\theta \right)/{\left(1-\exp\left(\theta \right)\right)}^{2}$. That is,

(A22) $$\begin{array}{cc} E\left(y\right)=b^{\prime }\left(\theta \right)=\frac{r\exp(\theta )}{1-\exp(\theta )}=\mu , & \\ V\left(y\right)=b^{\prime \prime }\left(\theta \right)a\left(\phi \right)=\frac{r\exp(\theta )}{{\left(1-\exp(\theta )\right)}^{2}}=\mu +\frac{\mu^{2}}{r}. \end{array}$$

To apply the GEE [70] and numerical optimization for searching the regression estimate for $\beta_{i}$ and *r*, since $r+\mu_{i}=r/\left(1-\exp\left(\theta_{i}\right)\right)$, one has $\frac{\mu_{i}}{r+\mu_{i}}=\exp\left(\theta_{i}\right)$. Then, consider to use log as the link function, one has $\theta_{i}=g\left(E\left[y_{i}\right]\right)=g\left(\mu_{i}\right)=\log\frac{\mu_{i}}{r+\mu_{i}}$ and variance function $V_{ii}=\mu_{i}+\mu_{i}^{2}/r$, then $g\left(\mu_{i}\right)=\log\frac{\mu_{i}}{\mu_{i}+r}=\sum_{j=0}^{p}x_{ij}\beta_{j}$ implies

(A23) $$\begin{array}{cc} \mu_{i}=r\frac{\exp(\sum_{j=0}^{p}x_{ij}\beta_{j})}{\left(1-\exp\left(\sum_{j=0}^{p}x_{ij}\beta_{j}\right)\right)}, & \\ V\left[E\left[y_{i}\right]\right]=r\frac{\exp(\sum_{j=0}^{p}x_{ij}\beta_{j})}{{\left(1-\exp\left(\sum_{j=0}^{p}x_{ij}\beta_{j}\right)\right)}^{2}}. \end{array}$$

From Equation (A23), one has

(A24) $$\begin{array}{cc} \frac{\partial \mu_{i}}{\partial \beta_{j}}=rx_{ij}\frac{\exp(\sum_{j=0}^{p}x_{ij}\beta_{j})}{1-\exp(\sum_{j=0}^{p}x_{ij}\beta_{j})}, & \\ \frac{\partial \mu_{i}}{\partial r}=\frac{\exp(\sum_{j=0}^{p}x_{ij}\beta_{j})}{1-\exp(\sum_{j=0}^{p}x_{ij}\beta_{j})}. \end{array}$$

Let $\beta =(\beta_{0},\beta_{1},\cdots ,\beta_{p})$, and one can deduce Equation (A23) in matrix form as in Equation (A25)

(A25) $$\left(\begin{array}{c} \frac{\partial \mu }{\partial \beta }\\ \frac{\partial \mu }{\partial r} \end{array}\right)V^{-1}\left(y-X\right)$$

In the phylogenetic comparative regression model context, Ref. [9] defined the variance-covariance between the observation with

(A26) $$V=\phi B^{1/2}CB^{1/2}$$

where C is the phylogenetic correlation matrix with elements $c_{ij}$ of the shared branch length of taxa *i* and *j* and B is a diagonal matrix with

(A27) $$B=\phi \cdot \mathrm{diag}\left(V\left(y_{i}|x_{i}\right)\right)=\phi \cdot \mathrm{diag}\left(\mu \right)=\phi \cdot \mathrm{diag}\left(\frac{r\exp(\sum_{j=0}^{p}x_{ij}\beta_{j})}{1-\exp(\sum_{j=0}^{p}x_{ij}\beta_{j})}\right).$$

Therefore, given the response $y={\left(y_{1},y_{2},\cdots ,y_{n}\right)}^{t}$ and the design matrix $X={\left(x_{0},x_{1},\cdots ,x_{p}\right)}^{t}$, by Equation (A12) the parameter estimate for β can be obtained by solving the nonlinear equation system of β. To incorporate C for deriving the phylogenetic negative binomial regression model, Equation (A12) can be expressed in a matrix form as shown in Equation (A28).

(A28) $$X_{(p+1)\times n}^{t}\mu_{n\times 1}1_{1\times n}^{t}{\left(B_{n\times n}^{1/2}C_{n\times n}B_{n\times n}^{1/2}\right)}^{-1}\left(Y_{n\times 1}-\mu_{n\times 1}\right)={\left[0\right]}_{(p+1)\times 1}.$$

The parameter estimate for β embedded in the μ vector with $\mu_{i}=r\exp\left(\sum_{j=0}^{p}x_{ij}\beta_{j}\right)/\left(1-\exp\left(\sum_{j=0}^{p}x_{ij}\beta_{j}\right)\right)$ can therefore be obtained through solving the system of the nonlinear equation of β.
